# RCC2 Expression Stimulates ER-Positive Breast Tumorigenesis

**DOI:** 10.1155/2020/5619462

**Published:** 2020-05-23

**Authors:** Weiqi Wang, Bing Xu, Zhaoxu Zhang, Kehua Fang, Xiaotian Chang

**Affiliations:** ^1^Medical Research Center of The Affiliated Hospital of Qingdao University, Wutaishan Road 1677, Qingdao, Shandong 266000, China; ^2^Department of Clinical Laboratory, The First Affiliated Hospital of Xi'an Jiaotong University, Xi'an, Shaanxi 710061, China; ^3^Medical Research Center of Shandong Qianfoshan Hospital, Shandong University, Jingshi Road 16766, Jinan, Shandong 250014, China; ^4^Medical Research Center, Shandong Provincial Qianfoshan Hospital, The First Affiliated Hospital of Shandong First Medical University, 16766 Jingshi Road, Jinan, Shandong 250014, China; ^5^Department of Neurology, Peking University People's Hospital, Beijing 100044, China; ^6^Clinical Laboratory of The Affiliated Hospital of Qingdao University, Wutaishan Road 1677, Qingdao, Shandong 266000, China

## Abstract

**Objective:**

Regulator of chromosome condensation 2 (RCC2) has been reported to be involved in the regulation of cell cleavage. This study investigated the effect of RCC2 expression on breast tumorigenesis.

**Methods:**

MCF-7 cells originating from estrogen receptor-positive (ER+) breast cancer were transfected with anti-RCC2 siRNA or RCC2-expressing plasmids. Cell proliferation, apoptosis, migration, and cytokine production in the transfected cells were examined using the CCK-8 assay, wound healing assay, and flow cytometry, respectively. PCR array was used to investigate the tumorigenic pathway of RCC2 in MCF-7 cells transfected with the anti-RCC2 siRNA. MCF-7 cells were also transfected with lentivirus-containing anti-RCC2 short hairpin RNA and were injected into BALB/c nude mice to generate tumor-bearing mice. Tumor growth in the mouse model was examined using magnetic resonance imaging by diffusion-weighted imaging analysis.

**Results:**

Western blotting and immunohistochemistry detected significantly increased expression of RCC2 in ER + breast tumor tissues compared with breast fibroadenoma samples. Inhibiting RCC2 expression decreased cell migration and stimulated apoptosis in MCF-7 cells, while overexpressing RCC2 stimulated cell migration and inhibited apoptosis. The inhibition of RCC2 expression significantly decreased breast tumor growth and IL-6 levels in the tumor-bearing mice. PCR array demonstrated that inhibiting RCC2 expression significantly decreased the expression of IGF1 and TWIST1, two well-known tumor-enhancing genes, in MCF-7 cells; conversely, overexpressing RCC2 increased the expression levels of these two genes in the transfected cells. This result was verified in the mouse model following inhibition of RCC2 expression in MCF-7 cells. Additionally, estradiol-17*β* suppressed MCF-7 cell apoptosis, stimulated cell proliferation and cell migration, and increased RCC2, IGF1, and TWIST1 expression. The siRNA-mediated inhibition of RCC2 expression alleviated the inhibitory effects of estrogen on apoptosis in MCF-7 cells, while overexpressing RCC2 enhanced the estrogen-driven inhibition of apoptosis. Modifying RCC2 expression had no impact on MCF-7 cell proliferation in the presence or absence of estradiol-17*β*.

**Conclusions:**

Our results suggest that estrogen-induced RCC2 expression prompts IGF1, TWIST1, and IL-6 expression, stimulates cell migration, and inhibits apoptosis to contribute to ER + breast tumorigenesis.

## 1. Introduction

Regulator of chromosome condensation-2 (RCC2), also known as telophase disc-60 kD (TD-60), is a member of the RCC family of guanine nucleotide exchange factors that comprise small GTPases. RCC2 is involved in the regulation of cell cleavage and is necessary for the proper completion of mitosis. During mitosis, RCC2 is required for correct assembly of the mitotic spindle and activation of key mitotic proteins [1–4]. Abnormal cell proliferation and apoptosis are essential characteristics of tumor tissues. RCC2 has been detected in lung, ovarian, colorectal, pancreatic, and gastric cancer [5–10]. Thus, investigating the tumorigenic mechanism of RCC2 is helpful for understanding tumor growth mechanisms.

In the present study, we analyzed RCC2 expression in breast tumor tissues. We also established a tumor-bearing mouse as the *in vivo* model and cell culture as the *in vitro* model to observe the effect of changed RCC2 expression on tumor growth. Furthermore, we used the RT^2^ Profiler™ PCR Array, a real-time PCR primer assay in a 96-well plate, to investigate the tumorigenic pathway of RCC2 in breast tumors. We aimed to understand how RCC2 is involved in tumorigenesis in breast cancer.

## 2. Materials and Methods

### 2.1. Tissue Collection

Tumor tissue specimens were collected at the Pathology Department of Tengzhou Central People's Hospital (Tengzhou, Shandong Province, China). Tumors were histologically diagnosed and pathologically classified according to the World Health Organization (WHO) classification system. All patients signed informed consent forms. This study was approved by the Ethics Committee of the Affiliated Hospital of Qingdao University (2019044).

### 2.2. Western Blot Analysis

Estrogen receptor+ (ER+) breast tumor tissues (*n* = 13, female; 34–64 years old, average 52 years old) and breast fibroadenoma tissues (*n* = 7, female; 17–59 years old, average 35 years old) were collected during duct removal surgery in the patients. Two hundred micrograms of tissue samples was individually homogenized in Cell Lysis Solution (Sigma, USA) and centrifuged at 12,000 × *g* for 30 min at 4°C. Thirty micrograms of total protein was separated by sodium dodecyl sulfate-polyacrylamide gel electrophoresis (SDS-PAGE) and then transferred to a polyvinylidene chloride membrane. The membrane was hybridized using anti-human RCC2 antibody (1 : 1000; Cell Signaling Technology; catalog number: 5104). Following three washes, the membrane was hybridized with sheep anti-rabbit antibody conjugated with peroxidase (1 : 1000; Sigma-Aldrich, USA). The immune signals of RCC2 protein were detected using Enhanced Chemiluminescence (ECL) kits (Millipore, USA). Quantitative analysis was performed using ImageQuant 5.2 software (General Electric Healthcare, USA). Another identical membrane was loaded with the same amount of protein sample and processed by the same protocol. This membrane was hybridized with anti-GAPDH antibody (catalog number: 10494-1-AP; Sanying, China) to normalize the sample loading.

The anti-TWIST1 antibody and the anti-insulin-like growth factor 1 antibody were obtained from Affinity (USA; catalog numbers: AF7945 and DF6096, resp.). Total proteins that were extracted from the MCF-7 cell line were analyzed using a similar protocol.

### 2.3. Immunohistochemistry

A breast tumor tissue array was commercially obtained from US Biomax (USA; catalog number: 483b). The array slide contained 40 breast invasive ductal carcinoma samples and 8 normal tissue samples. The tissue slide was incubated with the anti-RCC2 antibody overnight at 4°C. The immune signal of RCC2 was detected using an ultrasensitive kit (China Meixin Biology). The expression level of RCC2 in the tumor tissue sections was semiquantified by Chiew-Loon Koo's modified scoring system [11]. The system considers both staining intensity and stained area extent. The intensity of nucleic acid or cytoplasmic staining was scored as follows: no staining = 0, weak staining = 1, medium staining = 2, and strong staining = 3. The stained extent was scored as follows: 0% = 0, 1–10% = 1, 11–50% = 2, 51–80% = 3, and 81–100% = 4. The gene expression level was determined by multiplying the staining intensity score and the extent score. The minimum score of the gene expression level was 0, and the maximum score was 12.

### 2.4. Real-Time PCR Analysis

Total RNA was extracted from the transfected MCF-7 cells or tumor tissues. First-strand cDNA was synthesized by reverse-transcription using an RNA PCR Kit (TaKaRa, Japan). Real-time PCR was conducted using a ViiA7 DX Instrument (Life Technologies, USA). Relative mRNA expression was calculated using the comparative threshold cycle (C_t_) method. The target gene expression level was normalized to the expression level of GAPDH (glyceraldehyde-3-phosphate dehydrogenase). Forward primer and reverse primer sequences of the target genes were designed as follows: RCC2: 5′-AAGGAGCGCGTCAAACTTGAA-3′ and 5′-GCTTGCTGTTTAGGCACTTCTT-3′; GAPDH (glyceraldehyde-3-phosphate dehydrogenase): 5′-CAGAACATCATCCCTGCCTCTAC-3′ and 5′-TTGAAGTCAGAGAGGACCACCTG-3′; IGF1 (insulin-like growth factor 1): 5′-AGGATATTGGGCTTTACAACCTG-3′ and 5′-GAGGTAACAGAGGTCAGCATTTT-3′; SLIT2 (slit guidance ligand 2): 5′-GACTTCCAGAAGGTGCCGATGC-3′ and 5′-GTCATTGGTCCGTTGGTCACAGAG-3′; and TWIST1 (twist basic helix-loop-helix transcription factor 1): 5′-GACTTCCTCTACCAGGTCCTCCAG-3′ and 5′-TCCAGACCGAGAAGGCGTAGC-3′.

### 2.5. siRNA Interference

siRNA oligonucleotides targeting the RCC2 gene were designed as the sequence 5′-AACAGCAAGCTGCTTACCGCA-3′ and synthesized by QIAGEN. The cultured MCF-7 cells were transfected with the anti-RCC2 siRNA using HiPerFect Transfection Reagent (QIAGEN, Germany) according to the instructions of the manufacturer. Allstars siRNA that was provided with the kit has no target sequences in human genes and was used as a control.

### 2.6. RCC2-Expressing Plasmid Construction and Transfection

The full coding region of the RCC2 gene was obtained by PCR with the specific primers AscI-Fex (5′-TACTCAGGCGCGCCTGATGCCCAGGAAGAAGGCG-3′) and KpnI-Rex (5′-TACTCAGGTACCGAGGGTTCGGGGGTTGTA-3′). The resulting PCR product was verified using sequencing analysis and then inserted into the AscI/KpnI sites of a pcDNA 3.1 (+)-RFP expression vector. The RCC2-overexpressing plasmid was transfected into MCF-7 cells using PolyJet™ *In Vitro* DNA Transfection Reagent (SignaGen, USA) according to the instructions of the manufacturer.

### 2.7. Cell Proliferation Assay

The cultured MCF-7 cells were incubated for 2 h with Cell Counting Kit-8 solution (CCK-8, Dojindo, Japan). Absorbance was measured at 450 nm with a spectrophotometer (SpectraMax 190, Molecular Devices, USA).

### 2.8. Cell Apoptosis Assay

Apoptosis of MCF-7 cells was analyzed using flow cytometry (FACSAria II, BD Biosciences, USA). Annexin V-FITC (BioLegend, USA) and propidium iodide (PI) (BioLegend) were added to the cultured cells, and incubation was conducted at room temperature in the dark for 15 min.

### 2.9. Wound Healing Assay

MCF-7 cells were seeded in a 6 cm culture dish and cultured to >90% confluence. The cell monolayer was scratched with a p200 pipette tip to draw five parallel cell-free lines at the bottom of the plate (approximately 1 mm in width). The original medium was removed, and the cells were washed with PBS. Wound healing was imaged under a microscope at 1, 2, 3, and 4 days after scratching.

### 2.10. Estrogen-Stimulating Assay

MCF-7 cells (5 × 10^5^) were seeded in each well of 6-well plates and cultured overnight. Estradiol-17*β* (Solarbio, China) was dissolved in 0.1% ethanol and added to the culture medium. Cells treated with 0.1% ethanol served as a control.

### 2.11. Generation of Tumor-Bearing Mice

BALB/c (Vital River, China) nude mice (*n* = 5) were used to prepare the tumor-bearing mouse model. A lentiviral vector (LV3-GFP&Puro) containing a short hairpin RNA against RCC2 (shRCC2; target sequence 5′-AACAGCAAGCTGCTTACCGCA-3′) was purchased from GenePharma (Shanghai, China). MCF-7 cells were infected with this recombinant lentiviral vector. Cells transfected with the blank vector without the anti-RCC2 insert were used as a control. The infected MCF-7 cells (6 × 10^6^) were suspended in 200 *μ*L of PBS and subcutaneously injected into the right flanks of mice to generate tumor-bearing nude mice by a routine method. This experiment was performed in accordance with the Regulations for the Administration of Affairs Concerning Experimental Animals of China (2017). Animals were maintained under specific pathogen-free conditions and had access to water and a standard rodent diet. The mouse-borne tumors were measured every 4 days until 28 days after the cell injection. This study was approved by the Ethics Committee of The Affiliated Hospital of Qingdao University (2019044). This animal study was repeated three times.

### 2.12. Magnetic Resonance Imaging (MRI) Studies

MRI analysis of the tumor-bearing mice was performed using a small animal birdcage coil on a 3.0 Tesla MRI scanner (Discovery 750, GE Healthcare, USA). Diffusion-weighted imaging (DWI) analysis with a T2-weighted (T2W) spin-echo (SE) was used to measure the tumor physiological condition and tumor size in the mouse model. The apparent diffusion coefficient (ADC) was obtained by DWI analysis. High-signal regions (bright region) on DWI indicate clear dispersion of tumor tissues. A decrease in the ADC value indicates increased cell swelling and tissue density and decreased water content and distorted extracellular structures, which suggest vigorous proliferation and high activity of the tumor cells.

### 2.13. Assessment of Cytokine Production in Cell Culture and Tumor-Bearing Mice

Cytokine levels in the serum of tumor-bearing mice were measured using a Th1/Th2 analyte flow assay kit (Cell Gene, China). This assay can quantitatively measure the protein levels of interleukin-2 (IL-2), interleukin-4 (IL-4), interleukin-6 (IL-6), interleukin-10 (IL-10), tumor necrosis factor-*α* (TNF-*α*), and interferon-*γ* (IFN-*γ*) in a single blood sample. The analysis was performed using flow cytometry (NovoCyte D2040R; ACEA, USA).

### 2.14. Measuring IL-6 Levels Using ELISA

IL-6 levels in the serum of tumor-bearing mice were also measured using IL-6 ELISA kits (catalog number: ab100539; Abcam, USA) according to the manufacturer's instructions. The absorbance was immediately measured at 450 nm using a spectrophotometer (SpectraMax 190, Molecular Devices).

### 2.15. PCR Array Analysis

The cultured MCF-7 cells were transfected with siRNA targeting RCC2 (target sequence: 5′-AACAGCAAGCTGCTTACCGCA-3′) using HiPerFect Transfection Reagent (Qiagen). Cells transfected with Allstars siRNA were used as controls. The human breast cancer PCR array (Qiagen) was commercially obtained and was used to investigate the tumorigenic pathways of RCC2 in the cultured breast tumor cells. Total RNA that was extracted from the cultured cells was reverse-transcribed into first-strand cDNA as described above. The cDNA was mixed with an appropriate RT^2^ SYBR Green Master Mix (Qiagen) and was aliquoted into the wells of the RT^2^ Profiler PCR Array plate. The analysis of the PCR array was conducted using a ViiA7 DX (Life Sciences, USA). The relative expression level was determined by the ∆∆CT method. The raw array data were processed and analyzed using the PCR Array Data Analysis System at http://sabiosciences.com/pcrarraydataanalysis.php (Qiagen). Fold changes in expression level were calculated and expressed as log-normalized ratios of the siRNA-transfected cells and corresponding controls. Based on the manufacturer's instructions and our experience, those genes with at least a 3-fold change in the expression level were considered biologically significant in the study.

PCR array uses a fluorescence quantitative PCR method. All primers are verified in preliminary experiments to ensure that the results are consistent. The expression changes of 84 genes related to a certain signaling pathway or a certain biological function can be simultaneously detected on a 96-well plate. PCR array can be used to quantitatively analyze the expression of multiple genes related to a certain disease, signaling pathway, or biological process at the same time.

### 2.16. Statistical Analysis

Normality and homogeneity of variance tests were performed using SPSS 17.0 software (Biomedical Computer Programs, USA). Data that met the test criteria are presented as means ± SD. The significance of differences among multiple groups was analyzed using one-way analysis of variance (ANOVA). Comparisons between groups were performed using Fisher's least significant difference (LSD) method. Paired and/or unpaired Student's *t*-tests were used to evaluate the statistical significance of differences between two groups. *p* < 0.05 was considered statistically significant.

## 3. Results

### 3.1. Detecting RCC2 Expression in Tumor Tissues

**Figure 1:**
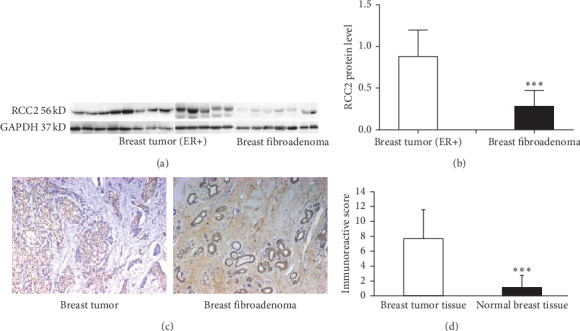
Expression of RCC2 in breast tumor tissues. (a) Western blot analysis detected an immunosignal with a molecular weight of 56 kDa in ER + breast tumor tissues (*n* = 13) and breast fibroadenoma (*n* = 7). The analysis also detected an immunosignal at a molecular weight of 37 kDa using anti-GAPDH in these tissue samples. (b) RCC2 expression was normalized with GAPDH expression. (c) Immunohistochemistry detected significant RCC2 expression in breast tumor tissues. The analysis also detected the expression of RCC2 in endothelial cells of normal breast tissues. Original magnification: 200x. (d) Semiquantification of RCC2 expression in breast tumor and healthy breast tissues. The *y*-axis indicates the level of RCC2 expression. ^*∗∗∗*^*p* < 0.001.

Western blot analysis was performed to detect RCC2 expression in ER + breast tumor tissues (*n* = 13) and breast fibroadenoma tissues (*n* = 7). The analysis revealed RCC2 expression at a molecular weight of 56 kDa in all 13 breast cancer samples and all 7 breast fibroadenoma samples (Figure 1(a)). Following normalization of RCC2 expression to GAPDH expression, there was a significant increase in RCC2 expression in these ER + breast tumor tissues compared with that in breast fibroadenoma tissues (*p*=0.004) (Figure 1(b)).

Immunohistochemistry was performed in a panel of breast tumor tissues to localize RCC2 expression. Thirty-two (93.7%) breast infiltrating ductal carcinoma samples showed significant RCC2 expression. The immunoreactive signal was located in the cytoplasm of the tumor cells. Three of eight (37.5%) normal breast tissue samples also presented immunostaining for RCC2, but the immune signal of RCC2 was limited to the epithelial cells of normal breast tissue, and the signal density was relatively lower in these tissues (Figure 1(c)). The immunoreactive score analysis indicated that breast invasive ductal carcinomas exhibited significantly increased RCC2 expression compared with that in the normal breast tissue samples (*p* < 0.001) (Figure 1(d)).

### 3.2. Determining the Effect of RCC2 Expression on Cell Activities of MCF-7 Cells

MCF-7 cells were transfected with anti-RCC2 siRNA for 4 days. Western blot analysis showed that RCC2 protein expression was significantly lower in MCF-7 cells transfected with anti-RCC2 siRNA than in the cells transfected with AllStars siRNA (*p*=0.019) (Additional file 1(a) and 1(c)). MCF-7 cells were also transfected with RCC2-expressing plasmids. Western blot analysis detected two RCC2 immunosignals in the transfected cells. The band at 86 kDa represented recombinant RCC2 protein conjugated to RFP, while the band at 56 kDa represented endogenous RCC2 protein. The analysis detected significantly more recombinant RCC2 protein in MCF-7 cells transfected with RCC2-expressing plasmids than in the cells transfected with blank RFP-expressing plasmids (*p* < 0.001) (Additional Files 1(b) and 1(d)).

**Figure 2:**
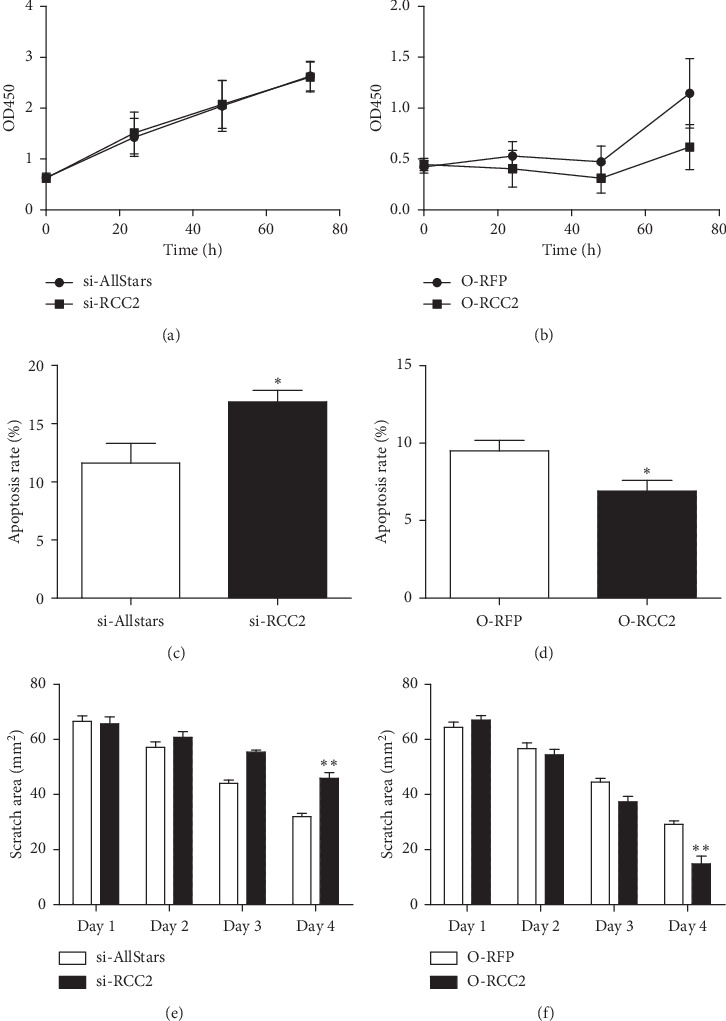
Effect of RCC2 expression on MCF-7 cells. MCF-7 cells were cultured and transfected with anti-RCC2 siRNA (si-RCC2) or RCC2-expressing plasmids (O-RCC2). AllStars siRNA (si-AllStars) and RFP-expressing plasmids (O-RFP) were used as the respective controls. (a, b) Cell proliferation was measured using the CCK-8 assay. (c, d) Apoptosis was measured using flow cytometry. (e, f) Cell migration ability was measured using a wound healing assay. ^*∗*^*p* < 0.05 and ^*∗∗*^*p* < 0.01.

The proliferation of MCF-7 cells was measured using the CCK-8 assay. No significant changes in cell proliferation were detected in MCF-7 cells transfected with anti-RCC2 siRNA compared to that of cells transfected with AllStars siRNA (*p*=0.957). The CCK-8 assay did not detect significantly changed cell proliferation in MCF-7 cells transfected with RCC2-expressing plasmids compared to that in cells transfected with blank vectors (*p*=0.149). The above observation indicated that RCC2 expression did not significantly affect MCF-7 cell proliferation (Figures 2(a) and 2(b)).

The effect of RCC2 expression on the apoptosis of MCF-7 cells was determined using flow cytometric analysis. The number of apoptotic cells was significantly increased in MCF-7 cells transfected with anti-RCC2 siRNA for 4 days compared with that in cells transfected with Allstars siRNA (*p*=0.037). When MCF-7 cells were transfected with RCC2-expressing plasmids for 4 days, the cell apoptosis was significantly decreased compared with that of tumor cells transfected with blank vectors (*p*=0.037). These observations demonstrated that RCC2 expression had a significant effect on apoptosis in MCF-7 cells (Figures 2(c) and 2(d)).

The cell migration ability of MCF-7 cells was measured using wound healing assays. On day 4 following transfection, cell migration was significantly less pronounced in MCF-7 cells transfected with anti-RCC2 siRNA than in cells transfected with AllStars siRNA (*p*=0.004). When MCF-7 cells were transfected with RCC2-expressing plasmids, their migration ability was significantly greater than that of the cells transfected with blank vectors (*p*_4day_=0.009). The above observation indicated that changed RCC2 expression significantly affected the migratory ability of MCF-7 cells (Figures 2(e) and 2(f)).

### 3.3. Determining Pathogenic Pathway of RCC2 in Breast Cancer

MCF-7 cells transfected with anti-RCC2 siRNA were subjected to Western blot analysis to examine the protein level of RCC2 in the cultured cells. RCC2 expression in the siRNA-transfected cells was much lower than that in the cells transfected with Allstars siRNA. The result is also shown in Additional Files 1(a) and 1(c). The human breast cancer PCR array detected significantly increased expression of SLIT2 (slit guidance ligand 2) and decreased expression of IGF1 (insulin-like growth factor 1) and TWIST1 (twist family BHLH transcription factor 1) with 3-fold changes in the expression levels (Additional File 2).

**Figure 3:**
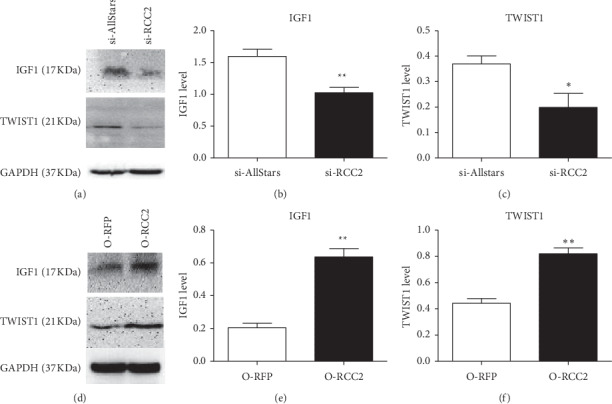
Assessment of the protein levels of IGF1 and TWIST1 in MCF-7 cells using Western blot analysis. MCF-7 cells were transfected with anti-RCC2 siRNA (si-RCC2) (a–c) or RCC2-expressing plasmids (O-RCC2) (d–f). Cells transfected with AllStars siRNA (si-AllStars) and RFP-expressing plasmids (O-RFP) were used as the corresponding negative controls. ^*∗*^*p* < 0.05, ^*∗∗*^*p* < 0.01, and ^*∗∗∗*^*p* < 0.001.

Real-time PCR was used to verify the result of the PCR array. The analysis did not detect significantly changed expression of SLIT2 (*p*=0.832) but detected decreased expression of IGF1 (*p*=0.013) and TWIST1 (*p*=0.019) in MCF-7 cells transfected with anti-RCC2 siRNA, which is partially in accordance with the results of the PCR array analysis. Moreover, real-time PCR detected significantly increased expression of IGF1, SLIT2, and TWIST1 in MCF-7 cells transfected with RCC2-expressing plasmids (*p*=0.044, 0.0273, and 0.013, respectively). The expression of SLIT2 was not consistent with the results of the PCR array and was not further investigated (Additional File 3). The above results were verified at the protein level using Western blotting. Significantly decreased IGF1 and TWIST1 expression were detected in MCF-7 cells transfected with anti-RCC2 siRNA (*p*=0.036 and *p*=0.007, respectively), while significantly increased IGF1 and TWIST1 expression were detected in the cells transfected with RCC2-expressing plasmids (*p*=0.002 and 0.003, respectively) (Figure 3).

### 3.4. Determining the Effect of RCC2 Expression on Tumor Growth in an Animal Model

**Figure 4:**
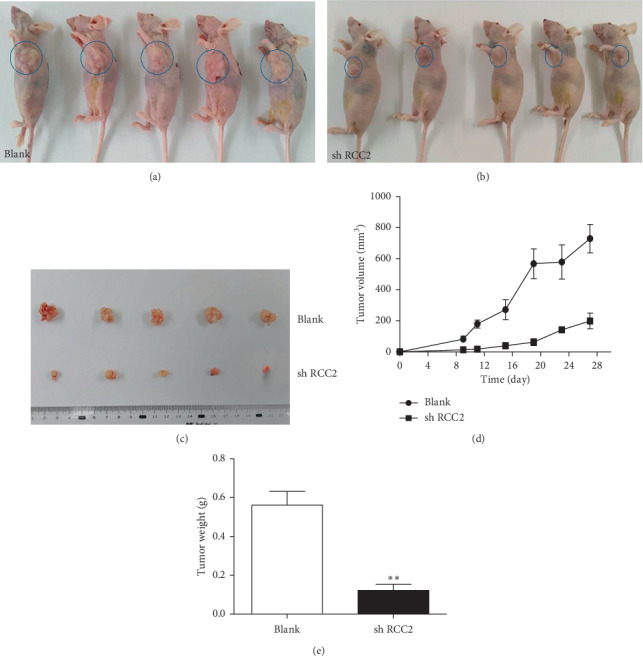
Effect of RCC2 expression on tumor growth in a xenograft mouse model. (a) Mice (*n* = 5) were injected with MCF-7 cells infected with lentivirus containing empty vector (blank). (b) Mice were injected with MCF-7 cells transfected with lentivirus containing anti-RCC2 shRNA (shRCC2) (*n* = 5). (c) Tumor tissues were dissected at 28 days after cell injection. (d) The tumor volume is recorded every 4 days and depicted on a map. (e) The tumor weight is depicted on a map.

MCF-7 cells were infected with lentiviral vector containing anti-RCC2 shRNA or an empty cassette. Real-time PCR and Western blotting detected significantly decreased mRNA and protein expression of RCC2 in the transfected cells (Additional File 4). MCF-7 cells with shRNA-mediated knockdown of RCC2 expression were injected into nude mice (*n* = 5) to generate tumor-bearing mice. Mice injected with anti-RCC2 shRNA-infected MCF-7 cells showed less tumor growth than did the mice injected with empty vector-infected cells. The average weight of the tumors from anti-RCC2 shRNA-infected MCF-7 cells was 0.123 g, and the average weight of the tumors resulting from the empty vector-infected cells was 0.562 g (*p*=0.001). The average tumor volume in mice injected with anti-RCC2 shRCC2-infected or empty vector-infected MCF-7 cells was 198.9 mm^3^ and 729.2 mm^3^, respectively (*p*=0.002) (Figure 4). Mouse tumors originating from anti-RCC2 shRNA-infected MCF-7 cells showed a higher DWI signal density than tumors from the empty vector-infected cells. The ADC value of tumor tissues generated from anti-RCC2 shRNA-infected MCF-7 cells was also significantly higher than that of tumors generated from empty vector-infected tumor cells (*p*=0.044) (Additional File 5).

The result of PCR array in MCF-7 cells was verified in this tumor-bearing mouse model. Real-time PCR detected decreased IGF1, RCC2, and TWIST1 expression in the tumors originating from MCF-7 cells infected with anti-RCC2 shRNA compared with that in tumors originating from cells infected with empty vector (*p*=0.004, *p*=0.008, and *p*=0.006, resp.) (Additional File 6).

Cytometric analysis was performed to quantitatively measure the levels of IL-2, IL-4, L-6, IL-10, TNF-*α*, and IFN-*γ* in the mouse serum. Mice injected with anti-RCC2 shRNA-infected MCF-7 cells showed significantly decreased levels of IL-6 (374.01 pg/ml) compared with those in mice injected with empty vector-infected MCF-7 cells (2153.10 pg/ml, *p*=0.005). There was no significant difference in the IL-2, IL-4, IL-10, TNF-*α*, and IFN-*γ* levels between blood samples from mice injected with anti-RCC2 shRNA-infected MCF-7 cells and samples from the controls (Additional Files 7(a) and 7(b)). An ELISA was performed to verify the IL-6 levels in the sera of the tumor-bearing mice. The IL-6 level was significantly decreased in mice injected with anti-RCC2 shRNA-infected MCF-7 cells compared with mice injected with empty vector-infected MCF-7 cells (Additional File 7(c)).

### 3.5. Determining the Effect of Estradiol-17*β* on RCC2 Expression

**Figure 5:**
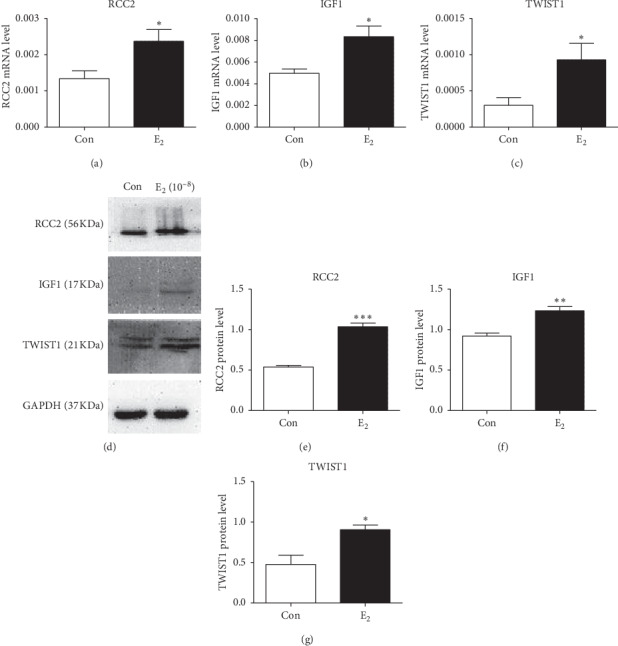
Expression levels of IGF-1, RCC2, and TWIST1 in MCF-7 cells treated with estradiol-17*β* (Ε_2_) at a concentration of 10^−8^ mol/L. Real-time PCR (a) and Western blot (b) were used to detect the transcriptional and translational levels of these targeted genes in MCF-7 cells, respectively. Cell culture in the presence of 0.1% ethanol was used as a control (con). ^*∗*^*p* < 0.05, ^*∗∗*^*p* < 0.01, and ^*∗∗∗*^*p* < 0.001.

MCF-7 cells were incubated with estradiol-17*β* at concentrations ranging from 10^−4^ to 10^−12^ mol/mL for 2 days. RCC2 showed significantly increased expression in cells treated with estrogen compared to that in cells treated with ethanol alone. The RCC2 gene exhibited the highest expression level in MCF-7 cells treated with estradiol-17*β* at a concentration of 10^−8^ mol/L (*p*=0.0021) (Additional File 8). Real-time PCR analysis detected significantly increased mRNA levels of RCC2, IGF1, and TWIST1 in the estradiol-17*β*-treated cells compared to those in cells treated with 0.1% ethanol (*p*=0.03, 0.018, and 0.046, resp.) (Figures 5(a)–5(c)). Western blot analysis also detected significantly increased RCC2 and IGF1 and TWIST1 protein expression in cells treated with estradiol-17*β* compared to that in cells cultured in the absence of estrogen (*p* < 0.001 and 0.002, respectively) (Figures 5(d)–5(f)). The TWIST1 antibody detected double bands. We performed semiquantification with the low and main bands and detected a significant increase in TWIST1 expression between the control and estrogen-treated samples. Furthermore, we detected a significant difference in the TWIST1 expression between the control and estrogen-treated samples in the upper band (*p*=0.016) (Figure 5(g)). Bands with a double molecular weight may be observed when the protein is generated through alternative splicing or cleavage during posttranslation. Interestingly, we did not find multiple bands of TWIST1 in MCF-7 cells that were not treated with estrogen.

**Figure 6:**
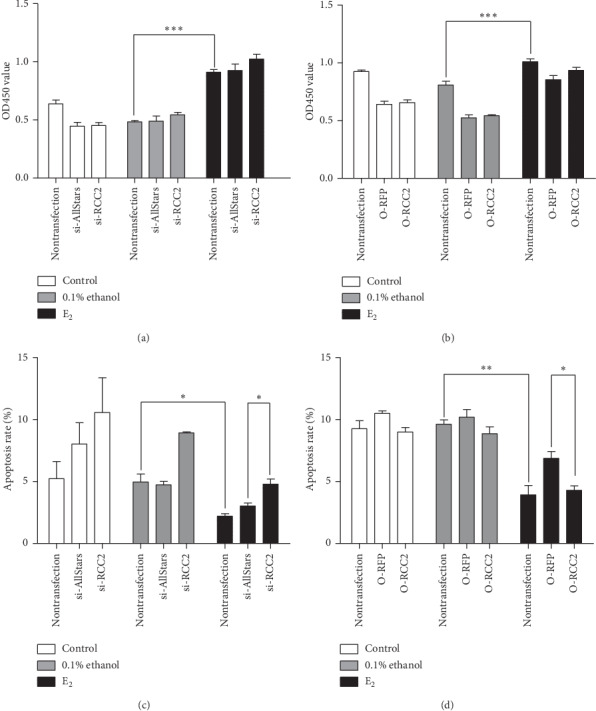
Effect of RCC2 expression on cell proliferation and apoptosis in MCF-7 cells cultured in the presence of estradiol-17*β* at a concentration of 10^−8^ mol/L. MCF-7 cells were transfected with anti-RCC2 siRNA (si-RCC2) or RCC2-expressing plasmids (O-RCC2). AllStars siRNA (si-AllStars) and RFP-expressing plasmids (O-RFP) were used as the corresponding controls. Untransfected cells were also used as a control. (a, b) Cell proliferation was measured using the CCK-8 assay. (c, d) Apoptosis was measured using flow cytometry. ^*∗*^*p* < 0.05, ^*∗∗*^*p* < 0.01, and ^*∗∗∗*^*p* < 0.001.

MCF-7 cells transfected with anti-RCC2 siRNA, RCC2-expressing plasmids, AllStars siRNA, or blank vectors were cultured in the presence of estradiol-17*β*. CCK-8 detected significant cell proliferation of MCF-7 cells treated with estradiol-17*β* (*p* < 0.001), indicating that estrogen treatment stimulates cell proliferation. However, compared with cells transfected with AllStars siRNA or blank vectors, MCF-7 cells transfected with anti-RCC2 siRNA or RCC2-expressing plasmids did not show a significant change in cell proliferation in presence of estrogen (*p*=0.200 and 0.101, respectively) (Figures 6(a) and 6(b)). These observations indicate that estrogen stimulates MCF-7 cell proliferation, but RCC2 expression has no effect on the cell proliferation in the presence or absence of estrogen. Flow cytometry detected significantly decreased apoptosis of MCF-7 cells in the presence of estrogen (*p*=0.014) compared with cells treated with 0.1% ethanol, indicating that estrogen treatment inhibits apoptosis. However, the apoptosis rate was significantly increased in MCF-7 cells transfected with anti-RCC2 siRNA compared with cells transfected with AllStars siRNA in the presence of estradiol-17*β* (*p*=0.02), and the addition of estrogen antagonized the stimulatory effect of knocking down RCC2 expression on apoptosis (*p*=0.003). The apoptosis rate was significantly decreased (*p*=0.019) in cells transfected with RCC2-expressing plasmids compared with that in cells transfected with the blank vectors in the presence of estradiol-17*β*. Furthermore, the addition of estrogen aggravated the inhibitory effect of overexpressing RCC2 on apoptosis (*p*=0.0001). These observations indicate that RCC2 expression inhibits apoptosis of MCF-7 cells, whether estrogen is present or not. Moreover, estrogen strengthened the inhibition of RCC2 expression on the cell apoptosis (Figures 6(c) and 6(d)).

## 4. Discussion

In recent years, RCC2 has been considered to be related to increased risk of many cancers [5–10], but there have been few reports on the effect of RCC2 on breast tumorigenesis. In the present study, we performed immunohistochemistry and detected extensive RCC2 expression in ER + breast tumor tissues. Western blotting confirmed the increase in RCC2 protein in ER + breast tumor tissues. Chen et al. also detected an increased mRNA level of RCC2 in breast tumor tissues by Gene Summary analyses with data from the Oncomine Online Database and The Cancer Genome Atlas (TCGA) Data Portal. They found that a high level of RCC2 was associated with a poor overall survival rate among breast cancer patients [12]. Gene, a database of NCBI, indicates that RCC2 is edited by a single copy of genomic gene in human genome and various human tumors (https://www.ncbi.nlm.nih.gov/gene/55920). Thus, we did not check RCC2 gene copy number in the study. Because MCF-7 cells originated from ER + breast tumor tissues, we transfected MCF-7 cells with an anti-RCC2 shRNA lentivirus vector to inhibit RCC2 expression. The cells with RCC2 knockdown were injected into nude mice to generate breast tumor-bearing mice. Compared with the tumors originating from blank vector-infected MCF-7 cells, tumors originating from anti-RCC2 shRNA-infected cells exhibited a decrease in the average weight from 0.562 g to 0.123 g and in the average tumor volume from 851.8 mm^3^ to 119.6 mm^3^ as well as a significant increase in the DWI and ADC values from 0.006 mm^2^/s to 0.0092 mm^2^/s. We repeated this experiment three times and obtained similar results. These findings demonstrated that inhibiting RCC2 expression significantly suppressed tumor growth. This is the first study demonstrating the importance of RCC2 for tumor growth in an animal model. This finding suggests that the expression of RCC2 is increased in ER + breast tumor tissues and contributes to tumor growth.

We transfected MCF-7 cells with anti-RCC2 siRNA or RCC2-expressing plasmids. No significant changes in cell proliferation were observed in MCF-7 cells transfected with anti-RCC2 siRNA or RCC2-expressing plasmid. On the other hand, the number of apoptotic cells was significantly increased in MCF-7 cells transfected with anti-RCC2 siRNA and significantly decreased in MCF-7 cells transfected with RCC2-expressing plasmids. MCF-7 cells transfected with anti-RCC2 siRNA showed low migration ability, but significantly increased migratory ability was observed in cells transfected with RCC2-expressing plasmids. This finding suggests that increased RCC2 expression in ER + breast tumor tissues contributes to tumor growth by inhibiting apoptosis and stimulating cell migration rather than by activating cell proliferation. Cell proliferation and apoptosis are two physiological phenomena that are closely linked but not consistently because they are regulated by different pathways. Pang et al. found that overexpression of RCC2 enhances cell motility and promotes tumor metastasis in lung adenocarcinoma by inducing epithelial-mesenchymal transition (EMT) [7]. Song et al. demonstrated that p53 binds to a palindromic motif in RCC2 promoter to regulate RCC2 expression to suppress cell migration and metastasis [6]. RCC2 acts in cell-migration machinery by connecting integrins [13]. Their reports were consistent with our finding on the effect of RCC2 expression on breast tumor cell activities. Involvement of RCC2 in apoptosis resistance could be of great interest. RCC2 is a highly conserved protein that structurally resembles the Ran guanine exchange factor (GEF) RCC1. There are few reports regarding RCC1 and RCC2 in apoptosis. Wu et al. found that RCC2 regulates apoptosis by blocking Rac1 signaling [5]. Chen et al. found that RCC2-mediated downregulation of the expression of survival proteins occurred via AKT and Bcl2 pathways [13].

Accumulating evidence indicates that endogenous estrogen plays a critical role in the development of breast cancer. Inhibiting estrogen activity is effective in preventing breast cancer in up to 50% of women with precancerous lesions [14]. We found that estradiol-17*β* increased RCC2 expression in cultured MCF-7 cells. Although estrogen itself stimulated the cell proliferation, MCF-7 cells did not show a significant change in proliferation when the cells were transfected with either anti-RCC2 siRNA or RCC2-expressing plasmids and cultured in the presence of estrogen. This result indicates that RCC2 expression is not involved in estrogen-mediated stimulation of cell proliferation. When MCF-7 cells transfected with anti-RCC2 siRNA were cultured in the presence of estradiol-17*β*, the rate of apoptosis was elevated compared to that in control cells transfected with AllStars siRNA, indicating that knocking down RCC2 expression alleviated the inhibitory effect of estrogen on apoptosis. When MCF-7 cells transfected with RCC2-expressing plasmids were cultured in the presence of estradiol-17*β*, the rate of apoptosis was decreased compared to that in the control cells transfected with blank vectors, indicating that overexpressing RCC2 heightened the inhibitory effect of estrogen on apoptosis. Our results not only correspond to the previous observation regarding the stimulatory effect of estrogen on breast tumor cells but also demonstrate that RCC2 expression strengthens the inhibitory effect of estrogen on apoptosis.

We employed a PCR array to investigate molecular pathway of RCC2 in breast cancer by subjecting MCF-7 cells transfected with anti-RCC2 siRNA. Based on the manufacturer's instructions and our experience, genes with at least a 3-fold change in the expression level were considered biologically significant in the analysis. The analysis indicated that there was significantly decreased expression of IGF1 and TWIST1 in MCF-7 cells with knocked-down RCC2 expression, which was verified in the transfected cells using real-time PCR and Western blot analyses. We also detected increased expression of these two genes in cells transfected with RCC2-expressing plasmids. Furthermore, in the tumor-bearing mouse model, we detected low levels of IGF1 and TWIST1 expression in tumors originating from MCF-7 cells transfected with anti-RCC2 shRNA. Increased TWIST1 and IGF1 expression were also detected in MCF-7 cells cultured in the presence of estradiol-17*β*. TWIST1 is a basic helix-loop-helix (bHLH) transcription factor that is overexpressed in a variety of tumors. Targeting TWIST1 or TWIST1-related molecules significantly inhibits tumor growth and cancer cell invasion and metastasis as well as reverses drug resistance, thus improving the survival of cancer patients [15]. TWIST1 expression in MCF-7 cells silenced the expression of Forkhead Box Protein A1 (Foxa1), which is concurrent with EMT induction, migration, invasion, and metastasis of the cells. TWIST1 expression is negatively correlated with Foxa1 in human breast tumors, and tumors with high TWIST1 and low Foxa1 expression are associated with poor distant metastasis-free survival [16, 17]. IGF1 is the primary mediator of growth hormone, and it thus has a strong influence on cell proliferation and differentiation and is a potent inhibitor of apoptosis. High serum concentrations of IGF1 are associated with an increased risk of breast, prostate, colorectal, and lung cancers. IGF1 underlies the actions of both estrogen and progesterone and has direct effects on mammary development and carcinogenesis [18]. Our results suggest that RCC2 plays a critical role in breast tumorigenesis by elevating IGF1 and TWIST1 expression to stimulate tumorigenesis.

This study detected decreased IL-6 levels in tumor-bearing mice that were injected with anti-RCC2 siRNA-transfected MCF-7 cells. IL-6 is a pleiotropic cytokine with known multiple functions in immune regulation, inflammation, and oncogenesis. Excessive IL-6 has been demonstrated in primary breast tumors and breast cancer patient sera and is associated with poor clinical outcomes in breast cancer. Deregulated overexpression of IL-6 induces cell proliferation, EMT, stem cell phenotype, angiogenesis, metastasis, cachexia, self-renewal of cancer stem cells, and therapeutic resistance in breast cancer [19–23]. IL-6 expression in gastric or breast cancer cells strongly enhanced tumor infiltration of TWIST1-expressing cancer-associated fibroblasts [24]. IL-6 has been reported to stimulate TWIST1 expression and EMT in MCF-7 cells [25]. The above reports support our findings on regulation of IL-6, TWIST1, and IGF1 by RCC2 expression. Our results suggest that increased RCC2 expression stimulates tumor growth by upregulating IGF1, TWIST1, and IL-6 expression.

RCC2 is located on 1p36.13 and is a member of the RCC family of guanine nucleotide exchange factors that comprise small GTPases. We recently detected a strong association of rs2244444 and rs12732894 in Rho guanine nucleotide exchange factor 10-Like (ARHGEF10L) locus with liver cancer. ARHGEF10L is also located in 1p36.13 and is a member of the RhoGEF family that promotes the active GTP-bound state of Rho GTPases. We simultaneously demonstrated that increased expression of ARHGEF10L stimulates hepatocellular tumorigenesis by activating the RhoA-ROCK1 (Rho-associated coiled-coil kinase-1)-phospho-ERM (phospho-Ezrin/Radixin/Moesin pathway) and EMT (epithelial-mesenchymal transition) in hepatocellular carcinoma [26].

## 5. Conclusion

The present study demonstrated increased expression of RCC2 in ER + breast tumor tissues. The increased expression of RCC2 stimulated cell migration and inhibited apoptosis, while inhibiting RCC2 expression reduced tumor growth in an *in vivo* animal model. RCC2 expression upregulated IL-6, IGF1, and TWIST1 expression both in animal models and in cultured ER + breast tumor cells. Estrogen increased RCC2 expression, which subsequently facilitated inhibition of the effects of estrogen on apoptosis and stimulation of cell migration. These results support the notion that RCC2 expression upregulated by estrogen induces IGF1, TWIST1, and IL-6 expression, stimulates cell migration, and inhibits apoptosis, which contributes to ER + breast tumorigenesis.

## Data Availability

All data supporting the conclusions of this work have been listed in this article.

## Supplementary Materials

Supplementary Materials Additional File 1: expression of RCC2 expression in MCF-7 cells as assessed by Western blot analysis. MCF-7 cells were cultured and transfected with (a) anti-RCC2 siRNA (si-RCC2) or (b) RCC2-expressing plasmids (O-RCC2). Additional File 2: determination of the downstream pathway of RCC2 in MCF-7 cells by means of human breast cancer PCR arrays. Additional File 3: assessment of the transcription levels of IGF1, SLIT2, and TWIST1 in MCF-7 cells using real-time PCR. MCF-7 cells were transfected with anti-RCC2 siRNA (si-RCC2) (a–c) or RCC2-expressing plasmids (O-RCC2) (d–f). Additional File 4: RCC2 mRNA and protein levels in MCF-7 cells as assessed by real-time PCR and Western blot analysis. Additional File 5: effect of RCC2 expression on tumor growth in tumor-bearing mice as assessed by MRI. Additional File 6: expression levels of IGF1, RCC2, and TWIST1 in tumors from xenograft mouse models as assessed by real-time PCR. Additional File 7: measurement of cytokine production in sera from tumor-bearing mice. Flow cytometric analysis was used to measure the IL-2, IL-4, Il-6, IL-10, TNF-*α*, and IFN-*γ* levels in the serum. Additional File 8: effect of estrogen on RCC2 expression. MCF-7 cells were incubated with estradiol-17*β* (*E*^2^) at concentrations from 10^−4^ to 10^−12^ mol/L.
