# Supplementary material for: RCC2 Expression Stimulates ER-Positive Breast Tumorigenesis
Source: J Oncol. 2020 May 23;2020:5619462. doi: 10.1155/2020/5619462 (PMC7262660; doi:10.1155/2020/5619462)
Supplement: Supplementary Materials — Additional File 1: expression of RCC2 expression in MCF-7 cells as assessed by Western blot analysis. MCF-7 cells were cultured and transfected with (a) anti-RCC2 siRNA (si-RCC2) or (b) RCC2-expressing plasmids (O-RCC2). Additional File 2: determination of the downstream pathway of RCC2 in MCF-7 cells by means of human breast cancer PCR arrays. Additional File 3: assessment of the transcription levels of IGF1, SLIT2, and TWIST1 in MCF-7 cells using real-time PCR. MCF-7 cells were transfected with anti-RCC2 siRNA (si-RCC2) (a–c) or RCC2-expressing plasmids (O-RCC2) (d–f). Additional File 4: RCC2 mRNA and protein levels in MCF-7 cells as assessed by real-time PCR and Western blot analysis. Additional File 5: effect of RCC2 expression on tumor growth in tumor-bearing mice as assessed by MRI. Additional File 6: expression levels of IGF1, RCC2, and TWIST1 in tumors from xenograft mouse models as assessed by real-time PCR. Additional File 7: measurement of cytokine production in sera from tumor-bearing mice. Flow cytometric analysis was used to measure the IL-2, IL-4, Il-6, IL-10, TNF-α, and IFN-γ levels in the serum. Additional File 8: effect of estrogen on RCC2 expression. MCF-7 cells were incubated with estradiol-17β (E2) at concentrations from 10−4 to 10−12 mol/L. [file 5619462.f1.pdf]

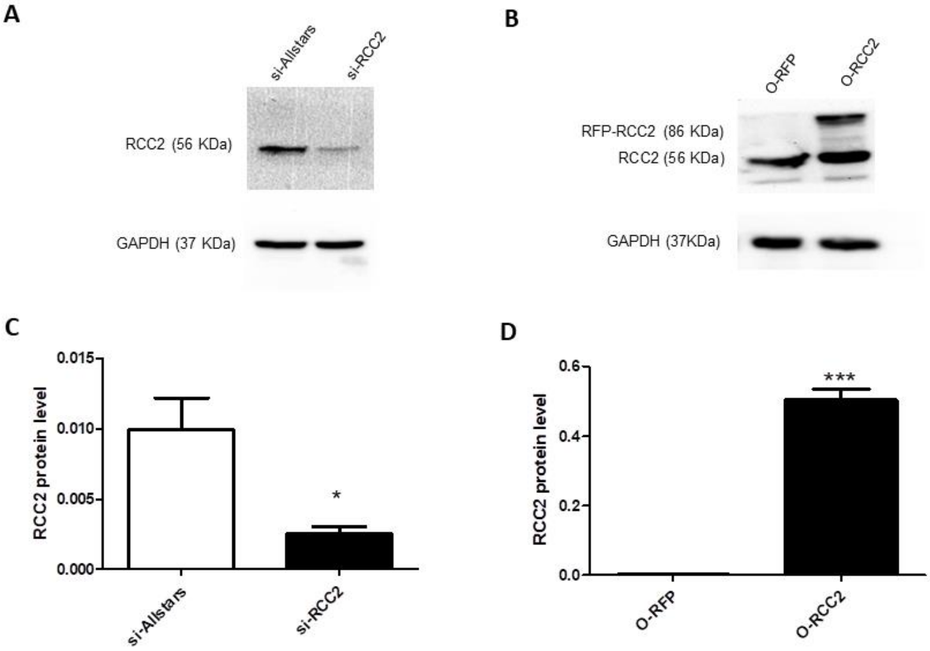


**Additional file 1.** Expression of RCC2 expression in MCF-7 cells as assessed by Western blot analysis. MCF-7 cells were cultured and transfected with (**A**) anti-RCC2 siRNA (si-RCC2) or (**B**) RCC2-expressing plasmids (O-RCC2). Allstars siRNA (si-Allstars) and RFP-expressing plasmids (O-RFP) were used as the respective controls. Following normalization by GAPDH expression, Western blot analysis detected a significant decrease (**C**) and increase (**D**) in RCC2 expression in MCF-7 cells transfected with anti-RCC2 siRNA or RCC2-expressing plasmid, respectively, compared with the corresponding controls. *p<0.05.

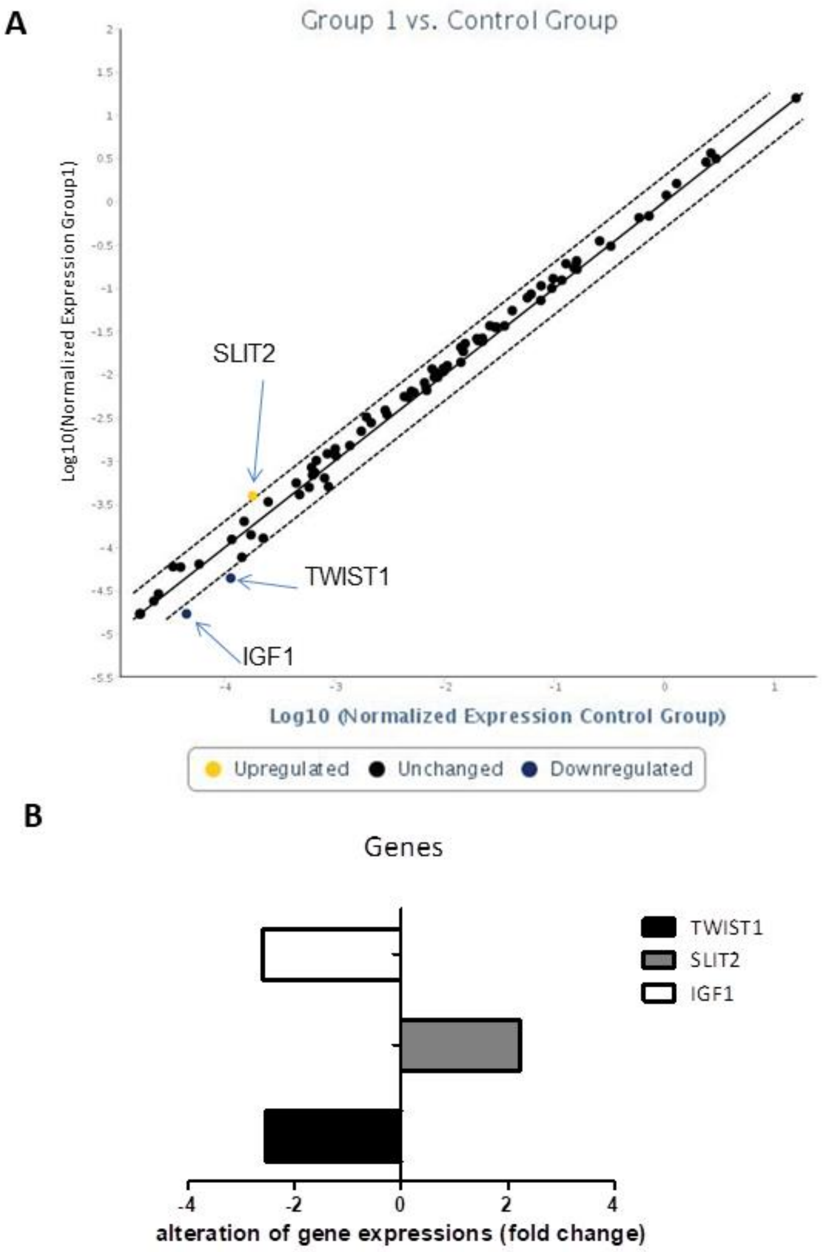


**Additional file 2.** Determination of the downstream pathway of RCC2 in MCF-7 cells by means of human breast cancer PCR arrays. (**A**) MCF-7 cells were transfected with anti-RCC2 siRNA, and cells transfected with Allstars siRNA were used as a negative control. Fold changes were calculated and expressed as log-normalized ratios

of the siRNA-transfected cells/controls. Genes with at least a 3-fold change in expression were considered to be biologically significant and are shown. (**B**) The result is depicted in a histogram.

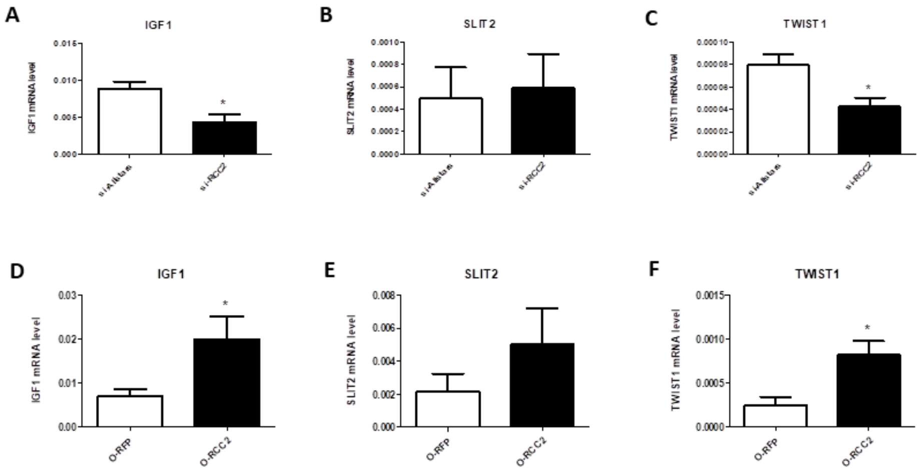


**Additional file 3.** Assessment of the transcription levels of IGF1, SLIT2 and TWIST1 in MCF-7 cells using real-time PCR. MCF-7 cells were transfected with anti-RCC2 siRNA (si-RCC2) (**A, B, C**) or RCC2-expressing plasmids (O-RCC2) (**D, E, F**). Cells transfected with Allstars siRNA (si-Allstars) and RFP-expressing plasmids (O-RFP) were used as the corresponding negative controls. *p<0.05, **p<0.01 and ***p<0.001.

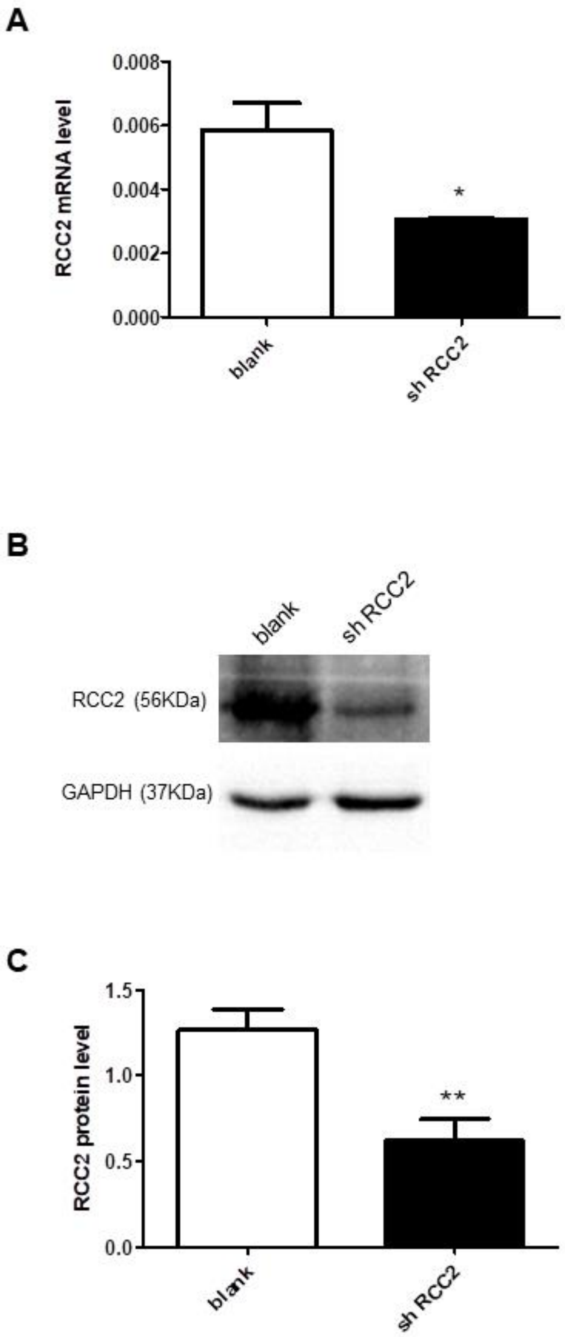


**Additional file 4.** RCC2 mRNA and protein levels in MCF-7 cells as assessed by real-time PCR and Western blot analysis. MCF-7 cells were infected with anti-RCC2 shRNA lentiviral vector (shRCC2) or empty lentiviral vector (blank). *p<0.05, and **p<0.01.

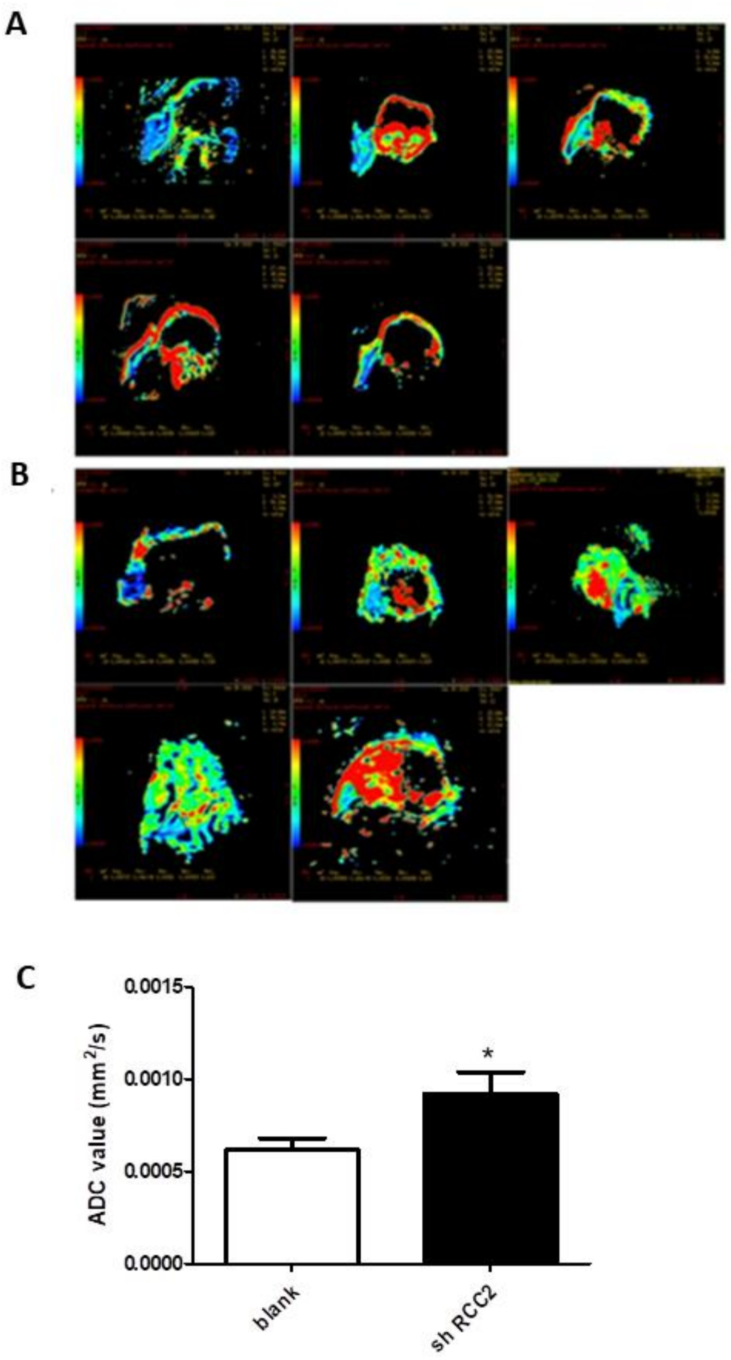


**Additional file 5.** Effect of RCC2 expression on tumor growth in tumor-bearing mice as assessed by MRI. (**A**) Mice injected with empty vector-infected MCF-7 cells were used as controls (blank). (**B)** MCF-7 cells infected with anti-RCC2 shRNA were injected into nude mice to generate tumor-bearing mice (shRCC2). Sagittal

T2-weighted MRI (T2WI) analysis of the tumors was conducted on the 28th day after injection. (**C**) The analytical result is presented in a bar graph. *p<0.05.

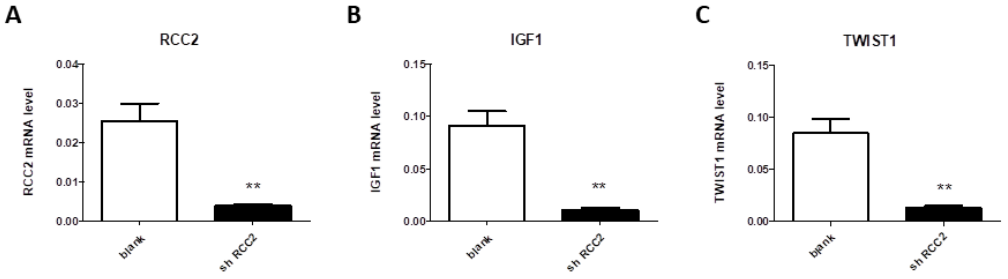


**Additional file 6.** Expression levels of IGF1, RCC2 and TWIST1 in tumors from xenograft mouse models as assessed by real-time PCR. The mice were injected with MCF-7 cells infected with lentivirus containing anti-RCC2 shRNA (shRCC2) or empty vectors (blank). *p<0.05.

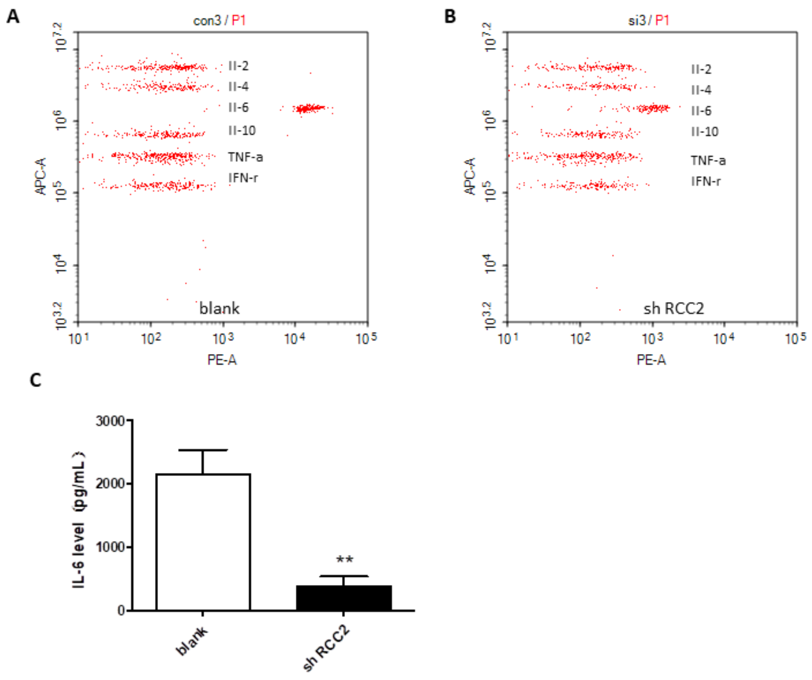


**Additional file 7.** Measurement of cytokine production in sera from tumor-bearing mice. Flow cytometric analysis was used to measure the IL-2, IL-4, Il-6, IL-10, TNF-α and IFN-γ levels in the serum. Serum was obtained from mice bearing tumors originating from MCF-7 cells infected with lentivirus containing anti-RCC2 shRNA (shRCC2) **(A)** or empty vectors (blank) **(B)**. (**C**) ELISA was used to measure the IL-6 levels in the serum. **$p<0.01$.

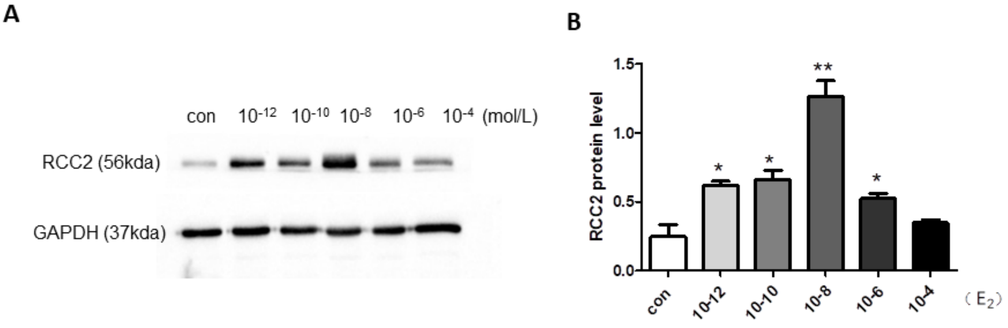


**Additional file 8.** Effect of estrogen on RCC2 expression. MCF-7 cells were incubated with estradiol-17β ($E_2$) at concentrations from $10^{-4}$ to $10^{-12}$ mol/L. RCC2 expression was detected using Western blotting (**A**), and the expression was normalized to GAPDH expression (**B**).
